# Identifying Patient Strengths Instruments and Examining Their Relevance for Chronic Disease Management: A Systematic Review

**DOI:** 10.5888/pcd18.200323

**Published:** 2021-04-29

**Authors:** Deshira D. Wallace, Ruchir N. Karmali, Christine Kim, Ann Marie White, Kurt C. Stange, Kristen Hassmiller Lich

**Affiliations:** 1 Gillings School of Global Public Health, University of North Carolina at Chapel Hill, Chapel Hill, North Carolina; 2 Northern California Kaiser Permanente Division of Research, Oakland, California; 3 University of Rochester Medical Center, Rochester, New York; 4 Children’s Institute, Rochester, New York; 5 Center for Community Health Integration, Case Western Reserve University, Cleveland, Ohio

## Abstract

**Introduction:**

Most health care focuses on patients’ deficits to encourage behavior change. A strengths-based approach, which relies on identifying patient strengths, has great potential to facilitate behavior change for chronic disease management. Little is known about instruments used to assess patient strengths. We conducted a systematic review to identify validated instruments that assess personal strengths by using a theory elaboration approach.

**Methods:**

We searched 8 databases including Web of Science, Cumulative Index of Nursing and Allied Health (CINAHL), and PsycINFO (through July 2019) to identify peer reviewed, English-language studies that described strength-based instruments. Thereafter, we evaluated the validity and reliability of the instruments according to 18 Scientific Advisory Committee of the Medical Outcome Trust (SACMOT) criteria, and used an inductive, iterative editing process to identify constructs measured by the instruments.

**Results:**

We identified 26 instruments that met our inclusion criteria. The instruments were validated in various clinical and nonclinical populations. Only 4 instruments met most of the SACMOT criteria for validation. We extracted 91 unique constructs that fell into 3 domains: inner strengths (49), external strengths (13), and personality constructs (29).

**Conclusion:**

A limited number of reliable and valid instruments are available to assess strengths for the adult population, particularly for clinical populations. Internal strengths can be leveraged to improve patient health; however, the development and validation of additional instruments to capture personal strengths is necessary to examine the multilevel influence of external strengths on individual behaviors and well-being.

**Summary**

**What is already known on this topic?**

Chronic disease management often focuses on what is wrong with patients rather than recognizing their strengths and resources. However, studies show the value of emphasizing personal strengths to improve outcomes.

**What is added by this report?**

How instruments evaluate personal strengths varies, making it especially difficult when determining the use of instruments in clinical populations. This systematic review defines the heterogeneity of constructs that research has used to examine personal strengths as well as the reliability and validity of strengths-related scales.

**What are the implications for public health practice?**

Understanding the value of these various scales can inform public health, and specifically primary care practice, to improve the care of adults managing 1 or more chronic conditions.

## Introduction

Approximately half of all US adults have 1 or more diagnosed chronic conditions, such as diabetes, heart disease, and arthritis (1). In 2016, the direct health care cost associated with chronic health conditions was $1.1 trillion dollars (2). Chronic conditions can be managed successfully by changing unhealthy behaviors (3–7). Patients, with their care team, can identify strategies and leverage skills to regulate behaviors (4). A strengths-based approach to chronic disease management can support self-management and behavior change.

The strengths-based approach emerged from the social work field and counteracts the deficit-based approach from the health science professions. A deficit-based approach focuses on what is wrong with patients rather than recognizing their strengths and resources (8). The strengths-based approach assumes that individuals have the capacity to grow, do the best they can, and know what is best for them (8). Strengths include personal attributes such as faith, use of humor, flexibility; interpersonal assets such as friends or family who can be called on for help; and external resources such as ability to access community resources for health. Whereas deficit-based approaches to chronic disease management focus on patients’ problems and behavioral shortcomings (eg, focusing on patient challenges in engaging with recommended behaviors), Rotegård et al (9) defined patient strengths, or health assets, as “the repertoire of potentials — internal and external strength qualities in the individual’s possession, both innate and acquired — that mobilize positive health behaviors and optimal health/wellness outcomes.” In practice, the care team works with a patient to identify their inherent strengths and the patient uses these strengths to promote recovery and well-being (10). The strengths-based approaches in counseling and case management are associated with an improvement in depressive symptoms, substance use behaviors, and postsurgery recovery by improving key determinants such as perceived patient empowerment (3,5,9,11–13).

Implementing a strengths-based approach relies on identifying a patient’s strengths (14–17). However, eliciting a patient’s strengths informally is challenging during time-constrained clinical visits (18). A formal strengths elicitation approach is needed to provide the structure during clinic visits to support patients in suggesting strengths to leverage (16). A concept analysis developed a theoretical framework for patient strengths (9); however, this framework was based on health assets in nursing care of cancer patients. Little is known about how patient strengths are operationalized as a construct in existing validated assessments — either broadly or among noninstitutionalized or community-based populations that frequent health care to manage a chronic condition (ie, clinical populations). Moreover, the construct of personal strengths has not been clearly defined in the literature, which could result in difficulties in distinguishing constructs from each other and instruments that may not adequately capture or sufficiently reflect the phenomenon.

The objective of our study was to identify and systematically summarize constructs that measure dimensions of personal strengths. Specifically, we were interested in understanding what we know about personal strengths and the extent to which instruments that measure personal strengths are validated for application in chronic care management. We reviewed instruments for measuring personal strengths by using the process of theory elaboration, to make a theoretical contribution in the field through specification of the aspects of the broad construct of personal strengths. The process of theory elaboration uses an existing model or conceptual idea as the basis for developing new theoretical insights, through contrasting, specifying, or structuring theoretical constructs to improve our understanding of the measurement of personal strengths (19). Although only a limited number of scales are specifically related to chronic disease management, examining currently validated scales that have been used in adults more broadly can provide information on what constructs and scales can be applied in populations that are managing chronic conditions. The results can inform the use of strengths-based scales in clinic settings for populations managing chronic conditions.

## Methods

Personal strengths is a broad construct; therefore, identifying dimensions of personal strengths can elucidate the multidimensionality of the construct. We used a theory elaboration approach for the analysis, specifically to improve construct validation and provide clarity of the scope of each dimension of personal strength as evidenced by previously conducted empirical studies (19).

This systematic review was conducted in accordance with the Preferred Reporting Items for Systematic Reviews and Meta-Analyses (PRISMA) guidelines (20). First, we conducted a search of peer-reviewed literature to identify validated instruments that operationalized strength constructs. Second, we extracted information from the instrument about constructs, reliability, and validity. Finally, we grouped constructs into categories defined by the health assets framework to understand at what level personal strengths (eg, inner, personality, external) are measured by the instruments (9). Personal strengths can be innate or acquired. Health assets are influenced by antecedents (eg, values, beliefs), an individual’s potential to pull from internal assets such as motivation, or external assets such as interpersonal support, to mobilize themselves to engage in positive health behaviors and improve their health (9).

### Data sources

We conducted the literature search to include any articles published from the earliest articles in each database through July 1, 2019. We worked with a university librarian to implement a search of peer-reviewed articles that combined phrases to describe *strengths* and *data collection instrument* in PubMed, Web of Science, Cumulative Index of Nursing and Allied Health (CINAHL), PsycINFO, PsycTESTS, Social Work Abstracts, the Health and Psychosocial Instruments, and Embase. After limiting results to humans and English-language articles and removing duplicates, the broad search was still explosive — returning more than 20,000 references. Therefore, we identified the strengths-related search terms with the worst specificity (ie, the largest absolute number of false positives based on a review of sampled abstracts for each term). The most problematic terms identified included “strength*”, “health resource*”, “protective factor*”, and “resilience”. Thereafter, we reviewed a randomly selected sample of 100 references and in working with the librarian, we replaced “strength*” in the main search with more specific terms capable of identifying appropriate references to balance a broad search with efficiency (ie, inner strength). Lastly, we reviewed any review papers (literature, scoping, systematic, meta-analysis) to further identify potentially relevant primary sources. Once we finalized our search strategy, we downloaded relevant citations to EndNoteX8 (Thomson ISI ResearchSoft), a reference management software. The search yielded 3,976 records.

### Criteria for study selection

We were interested in instruments that assessed strengths; therefore, we identified studies that met the following criteria: 1) measured strengths at the individual, interpersonal, or environmental level; 2) applied the instrument to an adult population; 3) presented reliability or validity information; 4) used a structured, self-reported questionnaire to assess strengths; 5) had instruments that comprised 3 or more strength-related dimensions (ie, constructs); and 6) were peer-reviewed and published in English before July 2019. We also included instruments developed in languages other than English with findings written in English. We did not include studies focused on child and adolescent samples because strengths may manifest differently across the developmental period. We also excluded studies if they either measured strengths as a subscale of a larger instrument or used qualitative instruments (eg, semi-structured interviews).

For screening we had 2 pairs of reviewers independently screen titles and abstracts. The full team met to resolve disagreements, reach consensus, and revisit the inclusion and exclusion criteria. If we could not reach consensus, the abstract was included for full-text review. Full-text review comprised 3 teams of 2 who closely assessed studies against the inclusion and exclusion criteria, with a focus on reported reliability and validity information for strengths constructs.

### Data extraction

We extracted descriptive characteristics, definitions of each construct, and reliability and validity information. For descriptive characteristics, we documented the purpose of the measure, the target population(s) in which the instrument was meant to be used (eg, gender-defined or clinically defined populations), and the settings in which the instrument was applied.

To assess reliability and validity information for each instrument we developed a structured extraction form by using the Scientific Advisory Committee of the Medical Outcome Trust (SACMOT) criteria (21). The 23 criteria are in 8 domains: conceptual models, reliability, validity, responsiveness, interpretability, burden, modes of administration, and cultural and language adaptations or translations (Table 1). Key validity criteria were face, content, criterion-related and construct validity, and reliability measures. Validity studies for each instrument were reviewed, and 2 reviewers (D.D.W. and R.N.K.) independently extracted validity data for each instrument by using the SACMOT criteria. Results were compared and any inconsistencies were resolved through team discussion. The first author reviewed all extraction for quality assurance.

**Table 1:** Criteria and Attributes for Reviewing Patient Strengths Instruments (N = 26)^a^

Attribute	Description	Review Criteria	No. of Instruments That Fit Criteria
**Conceptual and measurement model**	The rationale for and description of the concept and the populations that a measure is intended to assess and the relationship between these concepts	Rationale for and description of the concept to be measured	26
		Target population involvement in content derivation	8
		Information on dimensionality and distinctiveness of scales	26
		Rationale for deriving scale scores	7
**Reliability**	The degree to which an instrument is free from random error		5
Internal consistency	The precision of a scale, based on the homogeneity (intercorrelations) of the scale’s items at one point in time	Methods to collect reliability data	22
		Reliability estimates and standard errors for all score elements (classical test) or standard error of the mean over the range of scale and marginal reliability of each scale (modern Item Response Theory)	25
		Data to calculate reliability coefficients or actual calculations of reliability coefficients	21
Reproducibility	Stability of an instrument over time (test–retest) and inter-rater agreement at one point in time	Methods employed to collect reproducibility data	16
		Information on test-retest reliability and inter-rater reliability based on intraclass correlation coefficients	19
**Validity**	The degree to which the instrument measures what it purports to measure	Rationale supporting the particular mix of evidence presented for the intended uses	8
Content-related	Evidence that the domain of an instrument is appropriate relative to its intended use	Clear description of the methods employed to collect validity data	23
Construct-related	Evidence that supports a proposed interpretation of scores based on theoretical implications associated with the constructs being measured	Composition of the sample used to examine validity (in detail) Entails convergent validity, which measures whether 2 constructs that are theoretically related are actually related Discriminant validity measures if 2 constructs that should not be related are actually observed not to be related	22
Criterion-related	Evidence that shows the extent to which scores of the instrument are related to a criterion measure	Criterion measures data specified for each major population of interest	14
		Hypotheses tested and data relating to the tests	8
		Clear rationale and support for the choice of criteria measures	11
**Responsiveness**	An instrument’s ability to detect change over time	Longitudinal data that compare a group that is expected to change with a group that is expected to remain stable	7
**Interpretability**	The degree to which one can assign easily understood meaning to an instrument’s quantitative scores	Rationale for selection of external criteria of populations for purposes of comparison and interpretability of data	5
**Burden**	The time, effort, and other demands of the instrument		0
Respondent burden	The time, effort, and other demands placed on those to whom the instrument is administered	Information on 1) average and range of the time needed to complete the instrument, 2) reading and comprehension level, and 3) any special requirements or requests made of respondent	5
Administrative burden	The time, effort, and other demands placed on those who administer the instrument	Information about any resources required for administration of the instrument	2
**Modes of administration**	These include self-report, interviewer-administered, trained observer rating, computer-assisted interviewer-administered, performance-based measures	Information on the comparability of alternative modes	3
**Cultural and language adaptations or translations**	Involves 2 primary steps: 1) assessment of conceptual and linguistic equivalence, and 2) evaluation of measurement properties	Any significant differences between the original and translated versions	11

a Shortened version of key criteria based on Aaronson et al (21).

We created a table that included a row for every construct and the original definition from the source instrument to ensure fidelity throughout the extraction and synthesis process. Overlapping constructs were grouped together based on the similarity of their definitions. To organize the final table, we adapted the health assets framework developed by Rotegård and colleagues (9). Two authors (D.D.W. and R.N.K.) independently categorized each construct into the framework domains. Although the health assets framework distinguishes between assets (ie, strengths) and self-awareness, we considered self-awareness as a potential asset and coded constructs into this domain when appropriate. We developed emergent domains for constructs that did not fit into the existing framework. Finally, subdomains were created to reflect groups of constructs within domains. Unique constructs that were extracted from nonvalidated instruments were kept for the purposes of showcasing the diversity of strengths-related constructs and the gaps in validation.

## Results

Beginning with 3,976 articles and after removing 1,807 duplicates, 2,169 articles remained (Figure). We excluded 2,057 articles during title and abstract review for not specifying a focus on strengths-related predictors or outcomes, not being published in English, or using a child or adolescent sample only. During the full-text review, we excluded an additional 86 non-English, non–peer reviewed, or not strength-relevant studies. This review yielded 20 studies that met our inclusion criteria and underwent data extraction. Some studies had multiple strengths instruments, thereby producing 26 instruments for review. We extracted information about the populations in which these instruments were validated and sorted them (Table 2).

**Figure:**
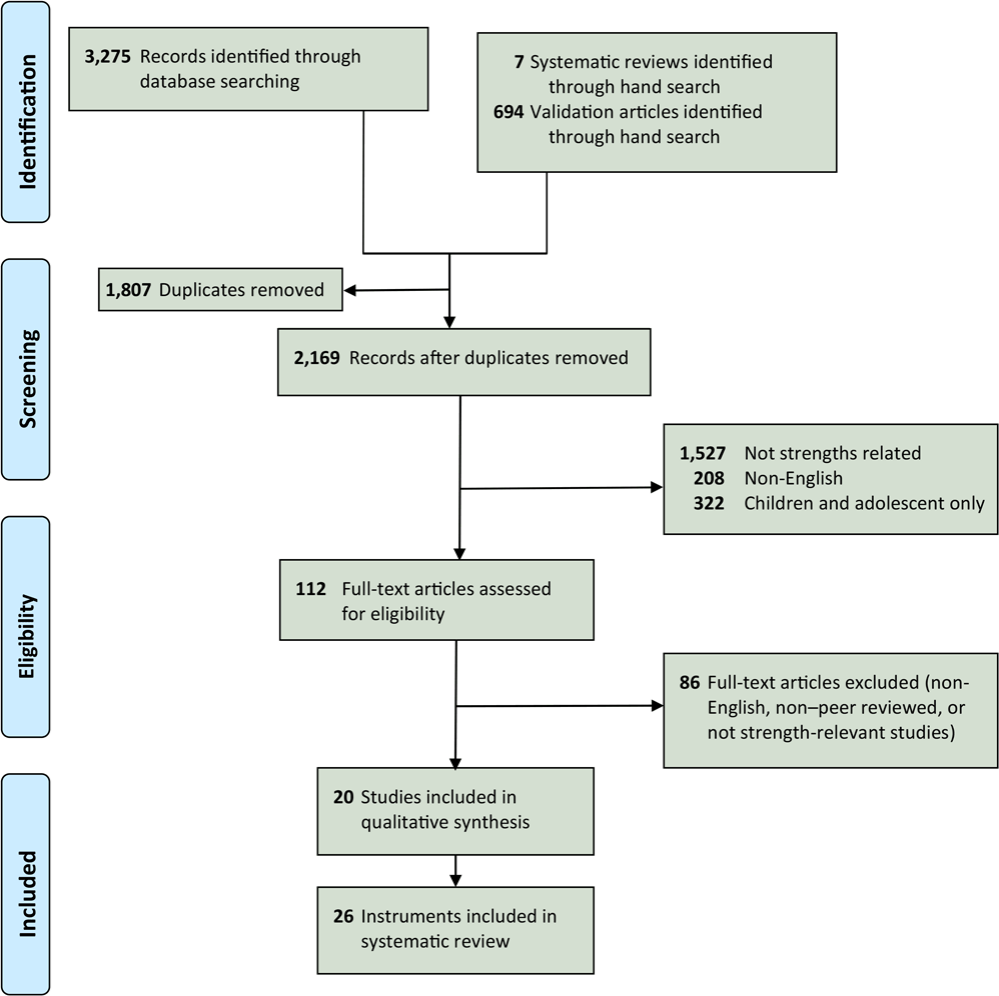
Preferred Reporting Items for Systematic Reviews and Meta-Analyses (PRISMA) flow diagram to obtain studies of strengths instruments to be analyzed for their relevance for chronic disease management.

**Table 2:** Description of Assessments of Patient Strengths Instruments^a^

No. and Assessment Name	Description	Populations Scale Was Applied	No. of Validity Articles	Year and Place Developed
Balanced Measure of Psychological Needs	Measures autonomy, competence, and relatedness.	College students in United States (Sheldon 2012)	1	2012, United States
Baruth Protective Factors Inventory	Measures resiliency.	Adults (Baruth 2002)	1	2001, United States
Big Five Personality	The Big Five Personality traits are extraversion, agreeableness, conscientiousness, emotional stability, and openness to experience, with each of these being measured on a continuum.	5 major occupational groupings: professionals, police, managers, sales, skilled/semi-skilled (Barrick 1991); Hispanic bilingual college students in Spain and United States (Benet-Martínez 1998); Italian adults (Fossati 2011); Dutch adults (Smits 2011) (Van Heck 1994); Estonian and Finnish adults (Pulver 1995); Polish adults (Strelau 1995); Japanese adults (Wada 1996); Kuwaiti adults (El-Ansarey 1997); college students in Brazil (Nunes 2007) (Hauck Filho 2012); German adults (Rammstedt 2007); Croatian adults (Mlacić 2008) (Hrebíčková 2010); Turkish adults (Karaman 2010) (Gençöz 2012) (Morsünbül 2014); Chinese college students (Huang 2010) (Wang 2010) (Shi 2015); Argentine adults (Ledesma 2011); Chinese female patients with breast cancer (Fan 2013)	38	1961, United States
Brief COPE	Measures coping with life stresses associated with a specific activity. Contains 3 subscales, with 2 of the 3 positively framed.	Nonstudent adult sample in United States (Carver 1997); French college students (Muller 2003) (Doron 2014); Swedish college students (Muhonen 2005); medical students in Malaysia (Yusoff 2010); caregivers of HIV-positive patients in Kenya (Kimemia 2011); adults with mild traumatic brain injury in US (Snell 2011); women with breast cancer in Mexico (Mejorada 2013); persons living with HIV/AIDS in southern India (Mohanraj 2015); people living with HIV/AIDS in China (Su 2015)	10	1989, United States
California Psychological Inventory	Measures personality and behavior under 4 domains: interpersonal style, intrapersonal style, achievement style, and stylistic modes.	College students in United States (Gough 1953) (Dicken 1963) (Darbes 1964); managers and supervisors in United States (Goodstein 1963) (Gough 1984); US Navy (Knapp 1963); Indian college students (Gough 1964); adults in France, Italy, Venezuela, and Turkey (Gough 1966); French adults (Chapuis 1970); Japanese college students (Nishiyama 1973) (Nishiyama 1975); Swedish delinquent and nondelinquent adults (Rosen 1974); multicountry delinquent and nondelinquent adults (Gough 1974); Israeli adults (Cohen 1977); Romanian adults (Pitariu 1980) (Pitariu 1981); Pakistani college students (Ahmed 1986); adults in Kuwait and Egypt (Torki 1988); Taiwanese adults (Ying 1991); US Coast Guard (Blake 1993); white-collar crime inmates in United States (Collins 1993); adults with alcoholism in United States (Kadden 1996); Norwegian college students (Sandal 2002)	55	1957, United States
Cancer Empowerment Questionnaire	Contains 6 factors; used to measure strengths that patients derive from themselves and their network: health care, social support, self-esteem, feeling connected, self-management, and community support.	Females with breast cancer in Korea (Shin 2015); female breast cancer survivors in Netherlands (van den Berg 2013) (Custers 2014)	3	2013, Netherlands
Caregiver Well-Being Scale	Two subscales: 1) basic needs subscale (4 factors: expression of feelings, attendance to physical needs, self-esteem, and security), and 2) activities of living (5 factors: time for self, maintenance of functions outside the home, family support, household maintenance, household tasks).	Medical informal caregivers in United States, mostly women (Tebb 1995) (Rubio 1999) (Berg-Weger 2000) (Rubio 2003) (Tebb 2013); social workers in United States (Rubio 2003); medical informal caregivers in Turkey (Demirtepe 2009)	6	1995, United States
Chronic Illness Resources Survey	Measures support and resources at each of the levels of the socioecological framework.	Patients with chronic conditions (Glasgow 2000), Spanish-speaking population (Eakin 2007)	3	2000, United States
College Student Hardiness Measure	Measures 3 factors of hardiness: control, commitment, and challenge.	Indian American college students (Atri 2007)	1	2007, United States
Connor-Davidson Resilience Scale	Developed to measure coping ability.	General population, primary care outpatient, psychiatric care outpatient in United States (Connor 2003); Korean adults (college students, nurses, firefighters) (Baek 2010); Spanish-speaking patients with fibromylagia (Notario-Pacheco 2014); Korean firefighters and rescue workers (Jeong 2015); German adults (Sarubin 2015); General Hong Kong population (Ni 2016); Chinese military (Xie 2016)	8	2003, United States
Dispositional Resilience Scale	Measures hardiness and includes dimensions of control, commitment, and challenge.	Norwegian armed forces employees (Hystad 2010); Italian adults (Picardi 2012); adults (Bartone 1989)	4 (plus 2 unpublished)	1989, United States
Five-Factor Personality Inventory	Assessed the Big Five factors of personality: extraversion, agreeableness, conscientiousness, emotional stability, and autonomy.	College students, faculty in United States (Hendriks 1999); internet users (Buchannan 2005); adults in Italy (Perugini 1998); male military recruits (Marshall 1994); Spanish adults (Salgudo 1997) (Rodríguez 2001); Dutch adults with personality disorders (Hendriks 1999); Turkish adults (Somer 2002) (Araz 2014); various European adult populations (De Fruyt 2004) (Sharma 2009); Indian adults (Sharma 2009)	13	1991, United States
Inner Strength Questionnaire	Developed to measure 4 dimensions for inner strength: knowing and searching, connectedness, physical self-spirit, and mental self-spirit.	Women with chronic illness in United States (Roux 2003) (Lewis 2011)	2	2003, United States
Interpersonal Support Evaluation List	Used to measure 4 conceptually distinct dimensions proposed to buffer the effects of stressful events: appraisal support, tangible assets support, belonging support, and self-esteem support.	College students (Brookings 1988) (Schonfeld 1991); homeless adults (Bates 1995) (Bates 1999); spinal cord–injured adults (McColl 1995) (Rintala 2013); HIV-infected adults in Venezuela (Bastardo 2000); Greek college students (Delistamati 2006); Polish adults (Szlachta 2009); battered women (Baumann 2012); Spanish-speaking college students (Mendoza 2012); Italian adults (Moretti 2012); Hispanic/Latino adults in United States (Merz 2014); adults by race (Sacco 2011)	14	1985, United States
Life Attitude Profile	Measures an individual’s attitude toward life and how they find meaning and purpose.	Canadian undergraduates (Reker 1981), Greek breast cancer patients (Anagnostopoulos 2011)	5	1981, Ontario, Canada
Mental Toughness Questionnaire (MTQ48)	The MTQ48 assesses total mental toughness and 4 subcomponents: challenge, commitment, confidence, and control. Translated into 14 languages. Used in occupational sector, education, health, and sports.	Adult athletes (Clough 2008) (Gucciardi 2012); university athletes (Sheard 2009); office management and administrative workers (Gucciardi 2012) (Perry 2013); general adult men (Perry 2013)	6	2002, United Kingdom
Norbeck Social Support Questionnaire	Measures social support.	Adults (graduate students in nursing) (Norbeck 1981); Spanish speakers (LaRoche 1994)	4	1980, United States
Post Traumatic Growth Inventory	Measures the general tendency to experience difficult events in a way that produces perceptions of benefits.	Undergraduate students with significant traumatic event in the past 5 years (Tedeschi 1996); cancer patients receiving palliative care (Mystakidou 2008); South American earthquake survivors (Leiva 2015); German adult stroke patients (Mack 2015); adult men who report sexual abuse during childhood (Saltzman 2015)	13	1996, United States
Psychological Capital Questionnaire	Measures hope, optimism, self-efficacy, and resilience.	Validated in the adult population in the United States and South Africa (Avey 2009); management students and engineers/technicians in the United States (Luthans 2009); Chinese workers (Wang 2012)	7	2004, United States
Psychological Well-being Questionnaire	Assesses individual’s well-being at a particular moment in time within 6 dimensions: autonomy, environmental mastery, personal growth, positive relationships with others, purpose in life, and self-acceptance.	Adult population aged 18 or older (Ryff 1995)	2	1989, United States
Resilience Scale for Adults	Measures 5 resilience factors among adults: personal strength, social competence, structured style, family cohesion, and social resources.	Adult outpatient clinic patients in Norway (Friborg 2003); military college students in Norway (Friborg 2005); university students in Norway (Friborg 2006); university students in Iran (Jowkar 2010); French-speaking Belgian college students (Hjemdal 2011); clinical and nonclinical college patients/students (Hilbig 2015); adults in Brazil (Hjemdal 2015)	7	2003, Norway
Resistance to Trauma Test	Measures 6 factors of personal strengths or resources: emotional intelligence and internal control; values, principles, and ethics; optimism, hope, and sense of humor; social skills and relationships; acceptance and adaptation; and internal congruency.	Spanish population who had experienced a traumatic event (Urra Portillo 2014)	1	2012, Spain
Response to Stressful Experiences Scale	5 protective factors: meaning-making and restoration, active coping, cognitive flexibility, spirituality, and self-efficacy.	Active-duty and reserve members of the military and veterans (Johnson 2011)	1	2011, United States
Sense of Coherence Scale	Based on Antonovsky’s salutogenesis framework. Used to measure 3 dimensions: comprehension, meaningfulness, and manageability.	33 languages in 32 countries (eg, Eriksson 2005)	51	1993, Israel
Solution-Focused Inventory	Way to measure people’s resources and their own resilience as a means of promoting a positive change.	Australian adult population (Grant 2012); Chinese college students (Yang 2015)	3	2011, Australia
Values in Action	Measures 24 character strengths (Macdonald 2008).	General adult population (Macdonald 2008); Spanish population (Azañedo 2014); Indian (Choubisa 2011); South African (du Plessis 2015); Chinese (Duan 2011) (Duan 2012); African (Khumalo 2008); Israeli (Littman-Ovadia 2008); Japanese (Otake 2005)	20	2004, United States

a A list of the publications cited in this table is in the Appendix.

Among the instruments assessed, 19 of 26 were developed in the United States, with the remaining 7 developed in Australia, Canada, Norway, Spain, Israel, the Netherlands, and the United Kingdom. Instruments were developed between 1957 and 2013. Although our method ensured all instruments were evaluated in at least 1 validity study, the number of validity studies ranged from 1 (eg, the Balanced Measure of Psychological Needs instrument [22]) to 55 (the California Psychological Inventory [23]), which is also the oldest instrument. Each instrument was validated for 1 or more populations (Table 2).

### Use in clinical populations

Among the 26 instruments that matched our inclusion criteria, 5 focused on clinical settings. For example, the Cancer Empowerment Questionnaire measures strengths that cancer patients and survivors derive from themselves and their social network (24,25). The Chronic Illness Resources Survey assesses support and resources at the individual, interpersonal, and community level (26). The Inner Strength Questionnaire was constructed and validated among women with chronic illnesses in the United States (27). The Resilience Scale for Adults was constructed for outpatient adults in Norway (28). Although 5 instruments were developed specifically for clinic populations, an additional 7 instruments were developed in nonclinic settings but later validated in various clinic populations, including the Big Five Personality (29), Brief COPE (30,31), Connor-Davidson Resilience Scale (32), Five-Factor Personality Inventory (33), Interpersonal Support Evaluation List (34), Life Attitude Profile (35,36), and Post-traumatic Growth Inventory (37,38). The Caregiver Well-being Scale assesses family caregivers’ strengths and domains where additional support is desirable for the caregiver (39).

### Scale construction, validity, and reliability assessment

We used 18 SACMOT criteria (21) to examine scale construction, reliability, and validity. These 18 criteria were reliability, reliability data collection, reliability estimates, reliability coefficient calculations, methods for reproducibility, test-retest or inter-rater reliability, validity rationale, content-related validity, construct-related validity, data on target population, hypothesis testing, criterion-related rationale, responsiveness, interpretability, respondent burden, administrative burden, administration modes, and cultural and language adaptations (Table 1). We found that the instruments with the most evidence of validity and reliability were the Big Five Personality (14 of 18 criteria), the California Psychological Inventory (13 of 18 criteria), the Resilience Scale for Adults (14 of 18 criteria), and the Sense of Coherence Scale (13 of 18 criteria) (40).

We found that definitions were clear for each construct, fulfilling the first SACMOT criterion (Table 1). All instruments had information on the dimensionality and distinctiveness of measured constructs. Most instruments (25 of 26) reported reliability estimates, such as a Cronbach α value (41).

Of the 26 instruments, 23 had at least 1 article detailing evidence on content validity, or the extent to which the items reflect the construct (42). Relatedly, construct validity measures comprised evidence for convergent and discriminant validity. We found 22 of 26 instruments assessed construct validity (eg, convergent and discriminant validity). Most instruments had measures of internal consistency to ensure reliability of the instrument and had reproducibility measures. Of the 26 instruments, 15 had information on less than 50% of the SACMOT criteria. The criterion with the least evidence was an evaluation of the administrative burden (2 of 26) and alternative modes of administering the instrument (3 of 26).

### Construct categorization

We found 91 distinct constructs (Table 3). Most instruments contained unique constructs, indicating that constructs did not conceptually overlap with each other across instruments. Common constructs across instruments included flexibility, spiritual strength, and autonomy.

**Table 3:** Inner, External, and Personality Strengths Constructs Found in Assessments of Patient Strengths Instruments

Domain	Construct	Source(s)	Definition
**Inner Strengths Constructs**			
**Relational strength**			
Perceived relations	Belonging support	Interpersonal Support Evaluation List	Perception of the availability of others for social interactions, and that one is a part of a group.
	Compensating experiences	Baruth Protective Factors Inventory	A sense that one’s informal networks provide opportunities or supplemental support systems above and beyond those in their formal networks.
	Love	Values in Action	The commitment to others or a commitment to what one does.
	Self-esteem support	Interpersonal Support Evaluation List	The perception that one has other people with whom one compares positively.
	Reward dependence^a^	Temperament and Character Inventory	Sentimentality, social sensitivity, attachment, and dependence on the approval of others. Characteristically sensitive, socially dependent, and sociable.
Perceived ability to get along with others	Consensuality	California Psychological Inventory	The perception of adhering to social norms when engaging in interpersonal interactions.
	Family cohesion	Resilience Scale for Adults	Having shared values, mutual appreciation, and support between family members.
	Relating to others	Balanced Measure of Psychological Needs Post Traumatic Growth Inventory	One’s feeling of more compassion and empathy for others after adversity such as trauma or loss. A feeling of connection or closeness with important others.
	Social competence (ie, positive relations)	Psychological Well-being Questionnaire Resilience Scale for Adults Resistance to Trauma Test	A general positive orientation, agreeableness, and sociability toward others.
	Social skills and relationships	Values in Action	An assessment of the behaviors that facilitate the interactions with family members, friends, colleagues, and acquaintances, as well as how one interacts with one’s environment.
	Cooperativeness^a^	Temperament and Character Inventory	The extent to which one conceives oneself as an integral part of human society. Characteristically tolerant, empathetic, compassionate, supportive, or principled.
Transcendence	Connectedness	Inner Strength Questionnaire	The connection to others such as family, society, and nature; having a spiritual dimension to life and being able to transcend oneself.
	Death acceptances	Life Attitude Profile	When a person transcends the fear of death.
	Gratitude	Values in Action	An inclination to acknowledge goodness in life and recognizing the source of goodness is outside of oneself.
	Spiritual strength	Connor-Davidson Resilience Scale Post Traumatic Growth Inventory Values in Action Brief COPE Response to Stressful Experiences Scale	Having an understanding of spiritual matters, coherent beliefs about the higher purpose and meaning in the universe. Knowing where one fits in the larger scheme, having beliefs about the meaning of life that shape conduct and provide comfort.
	Care^a^	Psychosocial Inventory of Ego Strengths	One’s inclination to take care of others, and commitment toward what one cares about and cares to do.
	Self-transcendence^a^	Temperament and Character Inventory	The extent to which one considers oneself as being an integral part of the universe as a whole. Characteristically spiritual, unpretentious, humble, or fulfilled.
**Motivational strength**			
Optimism	Hope	Values in Action Resistance to Trauma Test Psychological Capital Questionnaire	A belief and confidence that wishes will be obtained despite obstacles and barriers.
	New possibilities	Post Traumatic Growth Inventory	How one develops new interests, is future thinking, or appreciates new opportunities as they arise.
Meaning of life	Appreciation of life	Post Traumatic Growth Inventory Values in Action Measure of Personality Hardiness^a^ Mental Toughness Questionnaire (MTQ48)^a^	A greater understanding for one’s own personal values and meaning of life. Ability to recognize and admire the beauty in various areas of life such as art, science, and one’s own life.
	Meaningfulness	Sense of Coherence Scale Response to Stressful Experiences Scale^a^	One’s ability to find meaning in a situation.
	Purpose	Psychological Well-being Questionnaire Life Attitude Profile	A belief in the meaning of one’s life and one’s past and present actions.
Self-regulation	Goal orientation	Solution-Focused Inventory	Engaging with goal-setting and self-management behaviors.
	Personal growth	Psychological Well-being Questionnaire	A sense of self-improvement or personal expansion over time.
	Prudence	Values in Action	One’s inclination for far-sighted planning, short-term understanding, and goal-directed planning.
Self-efficacy	Self-efficacy	Psychological Capital Questionnaire Response to Stressful Experiences Scale	Confidence in one’s ability to be motivated to take action.
	Confidence	Mental Toughness Questionnaire (MTQ48)	Sense of self-belief and unshakeable faith considering one’s ability to achieve success.
**Protective strength**			
Protective strength	Active coping	Response to Stressful Situations Scale	An ability to engage in behaviors and thoughts that alter both internal and external sources of stress.
	Behavioral disengagement	Brief COPE	Ability to give up or reduce negative behaviors or stressors that may inhibit one’s ability to reach goals.
	Emotional stability	Big Five Personality Five-Factor Personality Inventory Solution-Focused Inventory Temperament and Character Inventory^a^	How one readily overcomes setbacks, disengages negative thoughts, and remains in the same mood in various situations.
	Emotional intelligence and internal control	Resistance to Trauma Test	Having the knowledge and ability to handle needs and control impulses for various situations.
	Having few stressors	Baruth Protective Factors Inventory	Number of stressors experienced in one’s life, with the assumption that those who are resilient experience fewer stressors.
	Trust, tolerance	Connor-Davidson Resilience Scale	One’s tolerance of negative affect or the strengthening effects of stress, as well as trust in one’s own instincts.
**Volitional strength**			
Independence	Autonomy	Balanced Measure of Psychological Needs Five-Factor Personality Inventory Psychological Well-being Questionnaire Life Attitude Profile	A sense of independence for choices and experiences of volition and self-regulation; feelings of personal agency.
	Control	Connor-Davidson Resilience Scale California Psychological Inventory Mental Toughness Questionnaire (MTQ48) Measure of Personality Hardiness^a^	A sense of control in life.
Determination	Bravery	Values in Action	Courage or ability to overcome fear, composed of cognitions, emotions, motivations, and decisions.
	Courage	Values in Action	One’s ability to use emotional strength to support and drive achievement, even when faced with adversity.
	Persistence	Values in Action	Maintenance of one’s behavior despite perceived barriers, frustration, and fatigue. Remaining determined and industrious.
	Firmness^a^	Inner Strength Questionnaire	Ability to take responsibility for oneself and others, and to deal with difficulties as they arise.
	Will^a^	Psychosocial Inventory of Ego Strengths	One’s ability to exercise free choice and demonstrate self-restraint and self-control.
Self-healing	Personal strength (resilience)	Cancer Empowerment Questionnaire Post Traumatic Growth Inventory Resilience Scale for Adults Resistance to Trauma Test General resilience^a^	The ability to bounce back from adversity, increased resiliency, and view of one’s current strengths as well as one’s belief in one’s ability to realize plans and goals.
	Physical self-spirit	Inner Strength Questionnaire	One’s ability to heal through activities, involvement, emotional honesty, and celebration.
**Self-awareness strength**			
Self-awareness	Coherence	Life Attitude Profile	A consistent understanding of life, others, and oneself.
	Comprehensibility	Sense of Coherence Scale	The ability to be aware and understand what is happening in the environment.
	Fidelity	Resistance to Trauma Test	An internalized preoccupation with being genuine or fair to oneself and others in terms of values, ethics, and beliefs.
	Internal congruency	Resistance to Trauma Test	How consistently one acts vis-à-vis how one thinks and what one intends.
	Mental self-spirit	Inner Strength Questionnaire	Ability to learn about one’s self.
	Perspective	Values in Action	One’s high level of knowledge and capacity to give and to recognize and weigh multiple sides before making decisions.
	Self-acceptance	Psychological Well-being Questionnaire	A positive attitude toward oneself and past choices.
**External Strengths Constructs**			
Social resources	Affect	Norbeck Social Support Questionnaire	The perception that the individual’s network gives love, respect, and admiration.
	Affirmation	Norbeck Social Support Questionnaire	The perception that others agree with the individual’s actions and serve as confidants.
	Aid	Norbeck Social Support Questionnaire	The perception that others are able to provide financial or physical help to complete tasks.
	Appraisal support	Interpersonal Support Evaluation List	The perceived availability of someone to talk to about important, personal issues.
	Social support	Cancer Empowerment Questionnaire Brief COPE Chronic Illness Resources Survey	Perceived support from those who are close to the individual, either in the form of information or emotions.
	Social resources	Baruth Protective Factors Inventory Cancer Empowerment Questionnaire Resilience Scale for Adults	One’s community or the type and quality of the social support and tangible or intangible resources received.
	Tangible assets support	Interpersonal Support Evaluation List	Perceived availability of material aid.
Institutional support	Community organizations	Chronic Illness Resources Survey	Having access to and participating in national and local organizations that support health. Examples include churches, employers, and other local volunteer organizations.
	Community supports	Chronic Illness Resources Survey	Having access to a community that supports health. Includes characteristics such as public transportation, community organizations that provide health information, and healthy food options.
	Employment support	Chronic Illness Resources Survey	Having an employer that supports health. Characteristics include flexible work schedules, access to workplace wellness facilities, and policies that support illness management.
	Health care support	Cancer Empowerment Questionnaire Chronic Illness Resources Survey	A perception of a good and collaborative relationship with health care staff as well as the ability to obtain medical information from health care staff.
	Media and policy resources	Chronic Illness Resources Survey	Extent that media and policy support chronic illness management. Includes health insurance coverage, medical costs, and positive sources of information regarding health from television, radio, billboards, and the internet.
	Neighborhood supports	Chronic Illness Resources Survey	Having access to an environment that supports health. Includes characteristics such as healthy food choice, safe parks, and friendly neighbors.
**Personality Constructs**			
**General personality traits**			
Intelligence	Intellectual strengths	Values in Action	One’s intellectual strengths, creativity, curiosity, and judgment, as well as a love for learning and appreciation of beauty.
	Wisdom	Values in Action	Positive reflection on one’s past and present, and a maturity in judgment.
Approach to interaction with others	Agreeableness	Big Five Personality Five-Factor Personality Inventory	Respect for others; ability to take others’ interests into account, and to make compromises.
	Extraversion	Big Five Personality Five-Factor Personality Inventory California Psychological Inventory	Being connected and sociable with others.
	Flexibility	Baruth Protective Factors Inventory Connor-Davidson Resilience Scale Resistance to Trauma Test Brief COPE California Psychological Inventory	One’s life trajectory and the openness to changing life circumstances as needed.
	Humility	Values in Action	An accurate self-assessment, recognition of limitations, and keeping accomplishments in perspective, as well as forgetting of the self.
	Leadership	Values in Action	The inclination to encourage a group of people and preserve harmony within groups.
	Teamwork	Values in Action	How one excels in a team, how dedicated one is in a team, how one shares and works hard for the success of the team.
Positivity	Humor	Values in Action Resistance to Trauma Test Brief COPE	Ability to make oneself and others laugh as well as provide a lighter perspective on events.
	Zest	Values in Action Measure of Personality Hardiness^a^ Mental Toughness Questionnaire (MTQ48)	One’s approach to all experiences with excitement and energy.
Justice	Fairness	Values in Action	An assessment of where one’s fairness falls under justice. One’s capacity to reason and make judgments.
	Forgiveness	Values in Action	Having kindness and compassion toward others, as well as one’s inclinations toward mercy and temperance.
	Honesty	Values in Action	A representation of the internal states, intentions, and commitments, both in public and private domains.
	Judgment	Values in Action	The ability to examine all aspects of a problem and weigh relevant evidence equally.
	Kindness	Values in Action	A belief that others are worthy of attention and affirmation for their own sake as human beings.
Approach toward learning and new experiences	Curiosity	Values in Action Big Five Personality Life Attitude Profile	Wanting to explore and learn about new topics or ideas; being open to new experiences.
	Creativity	Values in Action	The ability to think of new ideas or ways to do things and to influence the life course.
	Love of learning	Values in Action	The inclination to enjoy engaging with new information and skills.
	Novelty seeking^a^	Temperament and Character Inventory	Response to novel activities, impulsiveness to cues for rewards, and active avoidance of frustration. Characteristically quick-tempered, curious, easily bored.
**Resourcefulness**			
Self-management skills	Conscientiousness	Big Five Personality Five-Factor Personality Inventory	How one follows a routine and does things according to a plan.
	Manageability	Sense of Coherence Scale	One’s ability to manage one’s own situation either independently or with the help of important others.
	Planning	Brief COPE	Ability to think about how to address and create strategies to mitigate challenges in one’s life.
	Self-regulation	Values in Action	Ability to control and monitor one’ behaviors and emotions.
	Having a structured style	Resilience Scale for Adults	Being able to follow a routine, even if situations are challenging, as well as being organized and having clear goals and plans.
Ability to use resources	Resource activation	Brief COPE Solution-Focused Inventory Chronic Illness Resources Survey Response to Stressful Experiences Scale	The ability to determine solutions to problems or find resources to address problems.
	Self-directedness^a^	Temperament and Character Inventory	The extent to which one is responsible, reliable, resourceful, goal-oriented, and self-confident.
Knowledge about resources	Competence	Balanced Measure of Psychological Needs Connor-Davidson Resilience Scale	An application of one’s skills, abilities, and intelligence on the completion of a task. A demonstrated mastery of a skill or concept.
	Environmental mastery	Psychological Well-being Questionnaire	Ability and competence to manage one’s environment and external activities.
	Knowing and searching	Inner Strength Questionnaire	The ability to face potential diagnoses and subsequently to explore ways to use one’s strengths.

a These constructs are part of instruments that did not pass the validity assessment stage but had unique constructs not found in validated instruments.

We organized the constructs into 3 domains based on the health assets framework developed by Rotegård et al (9): inner strengths, external strengths, and personality constructs. Approximately half of the constructs were coded as inner strengths (n = 49). On the basis of the framework, inner strengths comprised constructs that measure how people relate to others and their environment (relational, n = 17), what drives people when they encounter challenging situations (motivational, n = 10), characteristics that buffer individuals from undesired health outcomes (protective, n = 6), self-determination to accomplish goals (volitional, n = 9), and self-reflective characteristics (self-awareness, n = 7).

Of 91 constructs, 13 were coded as external strengths, split into 2 domains: social resources and institutional support. Whereas social resources focus more on interpersonal resources, such as forms of tangible social support and aid (n = 7), institutional support contains constructs measuring community or institutional-level characteristics that could support positive behaviors, such as the presence of community organizations (n = 6).

We classified 29 constructs as personality, which related to innate individual traits. Most of these personality constructs came from well-established personality-based instruments such as the Big Five Personality or Five-Factor Personality Inventory (33). Through coding, personality constructs were further split into 2 subdomains. One was resourcefulness (n = 10), defined as the ability to perform tasks independently or seek help from others when necessary. Constructs coded to resourcefulness included those pertaining to self-management skills and knowledge of and ability to use resources. The second subdomain was general personality traits (n = 19) related to intelligence, justice, approach to interaction with others, positivity, and approaches to learning.

## Discussion

Personal strengths is a broad phenomenon comprising many constructs including internal strengths such as self-efficacy and personality or interpersonal strengths such as social support. Strengths can also come from community and social levels; however, we did not identify any validated scales focused on these higher-level strengths. The objective of this review was to identify and systematically summarize constructs that measure dimensions of personal strengths. This review shows evidence of a limited number of reliable and valid instruments available to assess strengths for the adult population. We identified 26 instruments, most of which were developed in the United States and had reliability estimates. Content validity and construct validity were the most documented forms of validity, and information on administrative burden was the least documented. The instruments with the most reliability and validity evidence were personality assessments (Big Five Personality) and perceptions of interpersonal reactions (California Psychological Inventory) and resiliency (Sense of Coherence Scale).

Furthermore, 91 constructs are represented across the 26 instruments. Over 85 percent of constructs focused on inner strengths or personality factors rather than external resources (ie, community assets). Most of the constructs were focused on internal resources, reflecting the overarching health care rhetoric and practice of self-management, which is focused on changing the individual without fully acknowledging the potential external assets that can be used to promote self-management behaviors (43,44). These results are consistent with a review conducted by Golden and Earp (45), which found that 95% of behavioral interventions are conducted at the individual level and 67% are conducted at the interpersonal level. Despite evidence showing that community-level and policy-level factors are more effective than individual-level factors to change health behaviors, very few interventions are done at these levels (46). Overall, the rich set of constructs identified as inner strengths or personality factors can be leveraged to improve patients’ health in primary care settings. Additional instruments focused on external assets can be developed to capture personal strengths and improve patient care more holistically.

The variability in validation studies may result from the high number of constructs used as proxies for personal strength; therefore, proper validity testing is necessary to advance the measurement of this broad construct. One specific type of validation that can help advance measurement is discriminant validity, which requires that measures of distinctly different constructs not be correlated with each other (42). Advancements in discriminating between the types of personal strengths can be made by further applying theory elaboration and construct proliferation techniques in future studies (19,47).

In considering the use of personal strengths to inform the treatment and management of chronic conditions for individuals, studies have found evidence of cultivating personal strengths to improve self-management behaviors and improve patient health outcomes (48–50). However, we found that the number of instruments developed specifically for clinical populations or validated in clinical populations is limited. This may reflect how providers may focus on treating problems and identifying risk factors rather than evaluating patients on their personal strengths that can be integrated as part of the treatment plan. Therefore, additional studies are needed to develop instruments to measure personal strengths in clinical populations. Relatedly, interventions are needed in health care settings to integrate validated instruments and relevant items from those instruments in clinical care to identify personal strengths and to start conversations about strengths-based approaches to improving chronic disease management. These interventions could target clinician awareness of instruments and knowledge of how to apply and interpret the results as a means of improving care.

### Limitations

Our systematic review found that few instruments assess strengths at the community and societal levels to emphasize an ecological approach to strengths identification. Most instruments were not developed specifically for clinical populations, therefore additional testing is needed in these populations. We limited our inclusion criteria to adults but recognize that validity assessments are needed for children and adolescents if they are to be implemented in clinical settings, particularly in primary care settings. Although we examined instruments validated in adult populations, the studies described participants homogenously rather than examining adults by different stages of the life course (eg, older adults). Parsing out adults by these key developmental periods could be explored given that health care is sorted by age-specific specialties, such as geriatrics. In addition, quality of life instruments were not included in this search as these assess multiple domains of an individual’s well-being. For this review, we wanted to limit the search to instruments that focus primarily on assessing personal strengths that an individual would have agency around, rather than instruments, such as health-related quality of life, that are broader constructs with a subdomain assessing strength. Our search may have been biased toward Western epistemologies, thereby excluding important strengths such as cultural group membership and connection, as research in Black psychology emphasizes (51).

### Future research

Resilience, an important characteristic all on its own, was not included in our literature search because of how large the search results became. In addition, many of the instruments were validated in the general adult population or college students. Primary care settings need strengths-based instruments that account for developmental differences across adulthood (eg, younger or older adults) and that reflect how strengths are manifested in different cultural, racial, or ethnic groups (eg, Black, Indigenous, Asian, White). To ensure the validity of these measures in different populations, additional psychometric testing is needed to determine if each construct has the same meaning for each group. Implementation research is needed on how to use these instruments in health care settings in a way that supports the workflow of clinicians and leverages patient-generated information as part of the treatment process. Future research could use the constructs identified here to develop a comprehensive instrument of patient strengths in the clinical setting to improve chronic disease management. These instruments must be validated across multiple clinical populations, including those managing multiple chronic conditions such as cancer, hypertension, and type 2 diabetes.

We have reviewed 26 reliable and valid instruments that measure personal strengths in clinical and nonclinical adult populations. Constructs in these instruments can be used in both research and clinic settings to improve self-management behaviors among people with chronic conditions. Although these instruments tap into different forms of strengths, few instruments assess external strengths (eg, interpersonal, community). The development and validation of additional instruments to capture personal strengths is necessary to examine the multilevel influence of external strengths on individual behaviors and well-being.

## Appendix

Following is a listing of studies cited in Table 2 of the article.

**Balanced Measure of Psychological Needs**

Sheldon KM , Hilpert JC . The balanced measure of psychological needs (BMPN) scale: an alternative domain general measure of need satisfaction. Motiv Emot 2012;36(4):439–51. 10.1007/s11031-012-9279-4

**Baruth Protective Factors Inventory**

Baruth KE , Carroll JJ . A formal assessment of resilience: the Baruth Protective Factors Inventory. J Individ Psychol (1998) 2002;58(3):235–44.

**Big Five Personality**

Barrick MR , Mount MK . The Big Five personality dimensions and job performance: a meta‐analysis. Pers Psychol 1991;44(1):1–26. 10.1111/j.1744-6570.1991.tb00688.x

Benet-Martínez V , John OP . Los Cinco Grandes across cultures and ethnic groups: multitrait-multimethod analyses of the Big Five in Spanish and English. J Pers Soc Psychol 1998;75(3):729–50. 10.1037/0022-3514.75.3.729 9781409

Fossati A , Borroni S , Marchione D , Maffei C . The big five inventory (BFI). Eur J Psychol Assess 2011;27(1):50–8. 10.1027/1015-5759/a000043

Smits IA , Dolan CV , Vorst HC , Wicherts JM , Timmerman ME . Cohort differences in Big Five personality factors over a period of 25 years. J Pers Soc Psychol 2011;100(6):1124–38. 10.1037/a0022874 21534699

Van Heck GL , Perugini M , Caprara G-V , Fröger J . The Big Five as tendencies in situations. Pers Individ Dif 1994;16(5):715–31. 10.1016/0191-8869(94)90213-5

Pulver A , Allik J , Pulkkinen L , Hämäläinen M . A Big Five personality inventory in two non–Indo‐European languages. Eur J Pers 1995;9(2):109–24. 10.1002/per.2410090205

Strelau J , Zawadzki B . The formal characteristics of behaviour — Temperament inventory (FCB — TI): validity studies. Eur J Pers 1995;9(3):207–29. 10.1002/per.2410090304

Wada S . Construction of the Big Five Scales of personality trait terms and concurrent validity with NPI. Shinrigaku Kenkyu 1996;67(1):61–7. 10.4992/jjpsy.67.61

El-Ansarey B . The psychometric properties of NEO five-factor inventory (NEO-FFI-S) based on the Kuwaiti society. Derasat Nafseyah 1997;7:277–310.

Nunes CHSS , Hutz CS . Development and validation of an Agreeableness scale in the Big Five personality model. Psicol Reflex Crit 2007;20(1):20–5. 10.1590/S0102-79722007000100004

Hauck Filho N , de Lara Machado W , Teixeira MAP , Bandeira DR . Evidencias de validade de marcadores reduzidos para a avaliacao da personalidade no modelo dos Cinco Grandes Fatores. Psicologia: Teoria e Pesquisa 2012;28(4):417–23. Portuguese. 10.1590/S0102-37722012000400007

Rammstedt B . The 10-item Big Five Inventory: norm values and investigation of sociodemographic effects based on a German population representative sample. Eur J Psychol Assess 2007;23(3):193–201. 10.1027/1015-5759.23.3.193

Mlacić B . National taxonomies, adjective markers and inventories: Three directions of application of the lexical approach to personality. Roczniki Psychologiczne 2008;11(1):152–60.

Hrebíčková M . Questionnaire in the Czech context. Stud Psychol (Bratisl) 2010;52:(3):165–77.

Karaman NG , Dogan T , Coban AE . A study to adapt the big five inventory to Turkish. Procedia Soc Behav Sci 2010;2(2):2357–9. 10.1016/j.sbspro.2010.03.336

Gençöz T , Öcül Ö . Examination of personality characteristics in a Turkish sample: development of Basic Personality Traits Inventory. J Gen Psychol 2012;139(3):194–216. 10.1080/00221309.2012.686932 24837020

Morsünbül Ü . The association of internet addiction with attachment styles, personality traits, loneliness and life satisfaction. Journal of Human Sciences 2014;11(1):357–72.

Huang Y , Wang L . Sex differences in framing effects across task domain. Pers Individ Dif 2010;48(5):649–53. 10.1016/j.paid.2010.01.005

Wang W , editor. How personality affects continuance intention: an empirical investigation of instant messaging. Proceedings of the Pacific Asia Conference on Information Systems (PACIS); 2010. No. 113.

Shi M , Liu L , Wang ZY , Wang L . The mediating role of resilience in the relationship between big five personality and anxiety among Chinese medical students: a cross-sectional study. PLoS One 2015;10(3):e0119916. 10.1371/journal.pone.0119916 25794003PMC4368674

Ledesma RD , Sánchez R , Díaz-Lázaro CM . Adjective checklist to assess the big five personality factors in the Argentine population. J Pers Assess 2011;93(1):46–55. 10.1080/00223891.2010.513708 21184330

Fan J , Zhu X-z , Tang L-l , Wang Y-p , Li L , Yang Y ,  Reliability and validity of Chinese Big Five Personality inventory brief version in breast cancer women. Chinese Journal of Clinical Psychology 2013;21(5):783–5.

**Brief COPE**

Carver CS . You want to measure coping but your protocol’s too long: consider the brief COPE. Int J Behav Med 1997;4(1):92–100. 10.1207/s15327558ijbm0401_6 16250744

Müller W. Lebe jetzt-einfach sein. Mainz (DE): Matthias-Grünewald-Verlag; 2003.

Doron J , Trouillet R , Gana K , Boiché J , Neveu D , Ninot G . Examination of the hierarchical structure of the Brief COPE in a French sample: empirical and theoretical convergences. J Pers Assess 2014;96(5):567–75. 10.1080/00223891.2014.886255 24579758

Muhonen T , Torkelson E . Kortversioner av frågeformulär inom arbets-och hälsopsykologi — om att mäta coping och optimism. Nord Psykol 2005;57(3):288–97. 10.1080/00291463.2005.10637375

Yusoff N , Low WY , Yip CH . Reliability and validity of the Brief COPE Scale (English version) among women with breast cancer undergoing treatment of adjuvant chemotherapy: a Malaysian study. Med J Malaysia 2010;65(1):41–4. 21265247

Kimemia M , Asner-Self KK , Daire AP . An exploratory factor analysis of the Brief COPE with a sample of Kenyan caregivers. Int J Adv Couns 2011;33(3):149–60. 10.1007/s10447-011-9122-8

Snell DL , Siegert RJ , Hay-Smith EJC , Surgenor LJ . Factor structure of the Brief COPE in people with mild traumatic brain injury. J Head Trauma Rehabil 2011;26(6):468–77. 10.1097/HTR.0b013e3181fc5e1e 21245767

Mejorada REO , Tufiño MAT , Sierra AV , Guerrero OT , Rosas AR , Sosa JJS . Afrontamiento en pacientes con cáncer de mama en radioterapia: análisis de la Escala COPE Breve. Psicología y Salud 2013;23(1):55–62.

Mohanraj R , Jeyaseelan V , Kumar S , Mani T , Rao D , Murray KR ,  Cultural adaptation of the Brief COPE for persons living with HIV/AIDS in southern India. AIDS Behav 2015;19(2):341–51. 10.1007/s10461-014-0872-2 25096895PMC4320041

Su XY , Lau JT , Mak WW , Choi KC , Feng TJ , Chen X ,  A preliminary validation of the Brief COPE instrument for assessing coping strategies among people living with HIV in China. Infect Dis Poverty 2015;4(1):41. 10.1186/s40249-015-0074-9 26370135PMC4570223

**California Psychological Inventory**

Gough HG . The construction of a personality scale to predict scholastic achievement. J Appl Psychol 1953;37(5):361–6. 10.1037/h0058511

Dicken CF . Convergent and discriminant validity of the California Psychological Inventory. Educ Psychol Meas 1963;23(3):449–59. 10.1177/001316446302300303

Darbes A , Mottesheard N , editors. A validity check of selected cluster scores of the California Psychological Inventory tests of 239 college students. Proceedings of the West Virginia Academy of Science 1964;36:191–4.

Goodstein LD , Schrader WJ . An empirically-derived managerial key for the California Psychological Inventory. J Appl Psychol 1963;47(1):42–5. 10.1037/h0043551

Gough HG . A managerial potential scale for the California Psychological Inventory. J Appl Psychol 1984;69(2):233–40. 10.1037/0021-9010.69.2.233

Knapp RR . Personality correlates of delinquency rate in a Navy sample. J Appl Psychol 1963;47(1):68–71. 10.1037/h0043494

Gough HG . Academic achievement in high school as predicted from the California Psychological Inventory. J Educ Psychol 1964;55(3):174–80. 10.1037/h0046186

Gough HG . Graduation from high school as predicted from the California Psychological Inventory. Psychol Sch 1966;3(3):208–16. 10.1002/1520-6807(196607)3:3<208::AID-PITS2310030305>3.0.CO;2-L

Chapuis C , Quintard G , Wourms J . A new personality inventory: the CPI (California Psychological Inventory). Bull Psychol 1970;24(16-18):997–1004.

Nishiyama T . Cross-cultural invariance of California Psychological Inventory. Psychologia 1973;16(2):75–84.

Nishiyama T . Validation of the CPI femininity scale in Japan. J Cross Cult Psychol 1975;6(4):482–9. 10.1177/002202217564010

Rosen A-S , Schalling D . On the validity of the California Psychological Inventory Socialization scale: a multivariate approach. J Consult Clin Psychol 1974;42(6):757–65. 10.1037/h0037566

Gough HG . Estimation of locus-of-control scores from the California Psychological Inventory. Psychol Rep 1974;35(1 Pt 2):343–8. 10.2466/pr0.1974.35.1.343 4438492

Cohen A , Farley FH . The common-item problem in measurement: effects on cross-cultural invariance of personality inventory structure. Educ Psychol Meas 1977;37(3):757–60. 10.1177/001316447703700318

Pitariu H , Helmine H . Investigarea personalităţii cu ajutorul Inventarului Psihologic California (CPI). Rev Psihol 1980;26(4):461–73.

Pitariu H . Validation of the CPI femininity scale in Romania. J Cross Cult Psychol 1981;12(1):111–7. 10.1177/0022022181121009

Ahmed I . Initial psychometric evaluation of URDU version of California Psychological Inventory (CPI). Pak J Psychol Res 1986;(June 22):3–16.

Torki MA . The CPI femininity scale in Kuwait and Egypt. J Pers Assess 1988;52(2):247–53. 10.1207/s15327752jpa5202_6

Ying Y-W . Validation of the California Psychological Inventory Femininity Scale in Taiwan college graduates. J Multicult Couns Devel 1991;19(4):166–72. 10.1002/j.2161-1912.1991.tb00553.x

Blake RJ , Potter EH 3d , Slimak RE . Validation of the structural scales of the CPI for predicting the performance of junior officers in the U.S. Coast Guard. J Bus Psychol 1993;7(4):431–48. 10.1007/BF01013757

Collins JM , Schmidt FL . Personality, integrity, and white collar crime: a construct validity study. Person Psychol 1993;46(2):295–311. 10.1111/j.1744-6570.1993.tb00875.x

Kadden RM , Litt MD , Donovan D , Cooney NL . Psychometric properties of the California Psychological Inventory Socialization scale in treatment-seeking alcoholics. Psychol Addict Behav 1996;10(3):131–46. 10.1037/0893-164X.10.3.131

Sandal GM , Endresen IM . The sensitivity of the CPI Good Impression Scale for detecting ‘faking good’ among Norwegian students and job applicants. Int J Sel Assess 2002;10(4):304–11. 10.1111/1468-2389.00220

**Cancer Empowerment Questionnaire**

Shin SH , Park H . [Development and validation of the empowerment scale for women with breast cancer]. J Korean Acad Nurs 2015;45(4):613–24. Korean. 10.4040/jkan.2015.45.4.613 26364536

van den Berg SW , van Amstel FKP , Ottevanger PB , Gielissen MFM , Prins JB . The cancer empowerment questionnaire: psychological empowerment in breast cancer survivors. J Psychosoc Oncol 2013;31(5):565–83. 10.1080/07347332.2013.825361 24010533

Custers JAE , van den Berg SW , van Laarhoven HWM , Bleiker EMA , Gielissen MFM , Prins JB . The Cancer Worry Scale: detecting fear of recurrence in breast cancer survivors. Cancer Nurs 2014;37(1):E44–50. 10.1097/NCC.0b013e3182813a17 23448956

**Caregiver Well-Being Scale**

Tebb S . An aid to empowerment: a caregiver well-being scale. Health Soc Work 1995;20(2):87–92. 10.1093/hsw/20.2.87 7544318

Rubio DM , Berg-Weger M , Tebb SS . Assessing the validity and reliability of well-being and stress in family caregivers. Soc Work Res 1999;23(1):54–64. 10.1093/swr/23.1.54

Berg-Weger M , Rubio DM , Tebb SS . The Caregiver Well-Being Scale revisited. Health Soc Work 2000;25(4):255–63. 10.1093/hsw/25.4.255 11103698

Rubio DM , Berg-Weger M , Tebb SS , Lee ES , Rauch S . Objectifying content validity: conducting a content validity study in social work research. Soc Work Res 2003;27(2):94–104. 10.1093/swr/27.2.94

Tebb SS , Berg-Weger M , Rubio DM . The Caregiver Well-Being Scale: developing a short-form rapid assessment instrument. Health Soc Work 2013;38(4):222–30. 10.1093/hsw/hlt019 24432489

Demirtepe D , Bozo Õ . Bakici ıyilik ölçeği’nin uyarlama, güvenirlik ve geçerlik çalişması. Turk Psikol Yaz 2009;12(23)28–37.

**Chronic Illness Resources Survey**

Glasgow RE , Strycker LA , Toobert DJ , Eakin E . A social-ecologic approach to assessing support for disease self-management: the Chronic Illness Resources Survey. J Behav Med 2000;23(6):559–83. 10.1023/A:1005507603901 11199088

Eakin EG , Reeves MM , Bull SS , Riley KM , Floyd S , Glasgow RE . Validation of the Spanish-language version of the Chronic Illness Resources Survey. Int J Behav Med 2007;14(2):76–85. 10.1007/BF03004172 17926435

**College Student Hardiness Measure**

Atri A , Sharma M , Cottrell R . Role of social support, hardiness, and acculturation as predictors of mental health among international students of Asian Indian origin. Int Q Community Health Educ 2006-2007;27(1):59–73. 10.2190/IQ.27.1.e 18039629

**Connor-Davidson Resilience Scale**

Connor KM , Davidson JR . Development of a new resilience scale: the Connor-Davidson Resilience Scale (CD-RISC). Depress Anxiety 2003;18(2):76–82. 10.1002/da.10113 12964174

Baek H-S , Lee K-U , Joo E-J , Lee M-Y , Choi K-S . Reliability and validity of the Korean version of the Connor-Davidson Resilience Scale. Psychiatry Investig 2010;7(2):109–15. 10.4306/pi.2010.7.2.109 20577619PMC2890864

Notario-Pacheco B , Martínez-Vizcaíno V , Trillo-Calvo E , Pérez-Yus MC , Serrano-Parra D , García-Campayo J . Validity and reliability of the Spanish version of the 10-item CD-RISC in patients with fibromyalgia. Health Qual Life Outcomes 2014;12(1):14. 10.1186/1477-7525-12-14 24484847PMC3922630

Jeong HS , Kang I , Namgung E , Im JJ , Jeon Y , Son J ,  Validation of the Korean version of the Connor-Davidson Resilience Scale-2 in firefighters and rescue workers. Compr Psychiatry 2015;59:123–8. 10.1016/j.comppsych.2015.01.006 25744698

Sarubin N , Gutt D , Giegling I , Bühner M , Hilbert S , Krähenmann O ,  Erste Analyse der psychometrischen Eigenschaften und Struktur der deutschsprachigen 10- und 25-Item Version der Connor-Davidson Resilience Scale (CD-RISC). Z Gesundh psychol 2015;23(3):112–22. 10.1026/0943-8149/a000142

Ni MY , Li TK , Yu NX , Pang H , Chan BH , Leung GM ,  Normative data and psychometric properties of the Connor-Davidson Resilience Scale (CD-RISC) and the abbreviated version (CD-RISC2) among the general population in Hong Kong. Qual Life Res 2016;25(1):111–6. 10.1007/s11136-015-1072-x 26198665

Xie Y , Peng L , Zuo X , Li M . The psychometric evaluation of the Connor-Davidson resilience scale using a Chinese military sample. PLoS One 2016;11(2):e0148843. 10.1371/journal.pone.0148843 26859484PMC4747584

**Dispositional Resilience Scale**

Hystad SW , Eid J , Johnsen BH , Laberg JC , Thomas Bartone P . Psychometric properties of the revised Norwegian dispositional resilience (hardiness) scale. Scand J Psychol 2010;51(3):237–45. 10.1111/j.1467-9450.2009.00759.x 20028488

Picardi A , Bartone PT , Querci R , Bitetti D , Tarsitani L , Roselli V ,  Development and validation of the Italian version of the 15-item dispositional resilience scale. Riv Psichiatr 2012;47(3):231–7. 2282543910.1708/1128.12446

Bartone PT . Predictors of stress-related illness in city bus drivers. J Occup Med 1989;31(8):657–63. 10.1097/00043764-198908000-00008 2668455

**Five-Factor Personality Inventory**

Hendriks AJ , Hofstee WK , De Raad B . The Five-Factor Personality Inventory (FFPI). Pers Individ Dif 1999;27(2):307–25. 10.1016/S0191-8869(98)00245-1

Buchanan T , Johnson JA , Goldberg LR . Implementing a five-factor personality inventory for use on the internet. Eur J Psychol Assess 2005;21(2):115–27. 10.1027/1015-5759.21.2.115

Perugini M , Ercolani AP . Validity of the five factor personality inventory (FFPI): an investigation in Italy. Eur J Psychol Assess 1998;14(3):234–48. 10.1027/1015-5759.14.3.234

Marshall GN , Wortman CB , Vickers RR Jr , Kusulas JW , Hervig LK . The five-factor model of personality as a framework for personality-health research. J Pers Soc Psychol 1994;67(2):278–86. 10.1037/0022-3514.67.2.278 7932063

Salgado JF . The Five Factor Model of personality and job performance in the European Community. J Appl Psychol 1997;82(1):30–43. 10.1037/0021-9010.82.1.30 9119797

Rodríguez-Fornells A , Lorenzo-Seva U , Andrés-Pueyo A . Psychometric properties of the Spanish adaptation of the Five Factor Personality Inventory. Eur J Psychol Assess 2001;17(2):145–53. 10.1027//1015-5759.17.2.145

Hendriks AJ , Hofstee WK , Raad BD . The five-factor personality inventory (FFPI). Pers Individ Dif 1999;27(2):307–25. 10.1016/S0191-8869(98)00245-1

Somer O , Korkmaz M , Tatar A . Bes Faktör Kisilik Envanteri’nin Gelistirilmesi-I: Ôlçek ve Alt Ôlçeklerin Olugturulmasi. Turk Psikol Derg 2002;17(49):21–33.

Araz A , Erkus A . Duygu Disavurum Tarzlarinin Kavramsallastirilmasi ve Ölçümü: Bir Envanter Gelistirme Çalismasi. Turk Psikol Derg 2014;29(74):50–61.

Fruyt FD , McCrae RR , Szirmák Z , Nagy J . The five-factor personality inventory as a measure of the five-factor model: Belgian, American, and Hungarian comparisons with the NEO-PI-R. Assessment 2004;11(3):207–15. 10.1177/1073191104265800 15358876

Sharma S , Deller J , Biswal R , Mandal MK . Emotional intelligence: factorial structure and construct validity across cultures. Int J Cross Cult Manag 2009;9(2):217–36. 10.1177/1470595809335725

**Inner Strength Questionnaire**

Roux G , Lewis K , Younger J , Dingley C . Development and testing of the inner strength questionnaire. J Cult Divers 2003;10(1):4–5. 12776541

Lewis KL , Roux G . Psychometric testing of the Inner Strength Questionnaire: women living with chronic health conditions. Appl Nurs Res 2011;24(3):153–60. 10.1016/j.apnr.2009.06.003 21777790

**Interpersonal Support Evaluation List**

Brookings JB , Bolton B . Confirmatory factor analysis of the Interpersonal Support Evaluation List. Am J Community Psychol 1988;16(1):137–47. 10.1007/BF00906076 3369379

Schonfeld IS , Ruan D . Occupational stress and preemployment measures of depressive symptoms: The case of teachers. J Soc Behav Pers 1991;6(7):95.

Bates DS . Cross-sectional and longitudinal analyses of two measures of social support among the homeless [dissertation]. Buffalo (NY): State University of New York at Buffalo; 1995.

Bates DS , Toro PA . Developing measures to assess social support among homeless and poor people. J Community Psychol 1999;27(2):137–56. 10.1002/(SICI)1520-6629(199903)27:2<137::AID-JCOP3>3.0.CO;2-B

McColl MA , Skinner H . Assessing inter- and intrapersonal resources: social support and coping among adults with a disability. Disabil Rehabil 1995;17(1):24–34. 10.3109/09638289509166624 7858278

Rintala DH . Predictive validity of social support relative to psychological well-being in men with spinal cord injury. Rehabil Psychol 2013;58(4):422–8. 10.1037/a0034357 24128267

Bastardo YM , Kimberlin CL . Relationship between quality of life, social support and disease-related factors in HIV-infected persons in Venezuela. AIDS Care 2000;12(5):673–84. 10.1080/095401200750003842 11218552

Delistamati E , Samakouri MA , Davis EA , Vorvolakos T , Xenitidis K , Livaditis M . Interpersonal Support Evaluation List (ISEL) — college version: validation and application in a Greek sample. Int J Soc Psychiatry 2006;52(6):552–60. 10.1177/0020764006074184 17294600

Szlachta E . The adaptation and preliminary validation of the Polish version of the Interpersonal Support Evaluation List (ISEL). Prz Psychol 2009;52(4):433–51.

Baumann M , Lurbe K , Leandro M-E , Chau N . Life satisfaction of two-year post-stroke survivors: effects of socio-economic factors, motor impairment, Newcastle stroke-specific quality of life measure and World Health Organization quality of life: bref of informal caregivers in Luxembourg and a rural area in Portugal. Cerebrovasc Dis 2012;33(3):219–30. 10.1159/000333408 22261643

Mendoza F , Bastardo Y . PIH68 quality of life and social support among college students in Venezuela. Value Health 2012;15(4):A204. 10.1016/j.jval.2012.03.1100

Moretti M , Simonelli A , Melloni M , Ronconi L . Interpersonal Support Evaluation List (ISEL): validation and application in an Italian sample. Psicol Soc 2012;(3):447–57. 10.1482/38446

Merz EL , Roesch SC , Malcarne VL , Penedo FJ , Llabre MM , Weitzman OB ,  Validation of Interpersonal Support Evaluation List-12 (ISEL-12) scores among English- and Spanish-speaking Hispanics/Latinos from the HCHS/SOL Sociocultural Ancillary Study. Psychol Assess 2014;26(2):384–94. 10.1037/a0035248 24320763PMC4048059

Sacco E , Bientinesi R , Marangi F , D’Addessi A , Racioppi M , Gulino G ,  [Overactive bladder syndrome: the social and economic perspective]. Urologia 2011;78(4):241–56. Italian. 10.5301/RU.2011.8886 22237808

**Life Attitude Profile**

Reker G , Peacock E . The Life Attitude Profile (LAP): a multidimensional instrument for assessing attitudes towards life. Can J Behav Sci 1981;13(3):264–73. 10.1037/h0081178

Anagnostopoulos F , Slater J , Fitzsimmons D , Kolokotroni P . Exploring global meaning in Greek breast cancer patients: validation of the Life Attitude Profile — Revised (LAP-R). Psychooncology 2011;20(4):419–27. 10.1002/pon.1755 20878848

**Mental Toughness Questionnaire (MTQ48)**

Clough P , Earle K , Strycharczyk D . Developing resilience through coaching: MTQ48. In: Passmore J, editor. Psychometrics in coaching: using psychological and psychometric tools for development. London (GB): Kogan Page; 2008. p. 208–23.

Gucciardi DF , Hanton S , Mallett CJ . Progressing measurement in mental toughness: a case example of the Mental Toughness Questionnaire 48. Sport Exerc Perform Psychol 2012;1(3):194–214. 10.1037/a0027190

Sheard M , Golby J , Van Wersch A . Progress toward construct validation of the Sports Mental Toughness Questionnaire (SMTQ). Eur J Psychol Assess 2009;25(3):186–93. 10.1027/1015-5759.25.3.186

Perry JL , Clough PJ , Crust L , Earle K , Nicholls AR . Factorial validity of the mental toughness questionnaire-48. Pers Individ Dif 2013;54(5):587–92. 10.1016/j.paid.2012.11.020

**Norbeck Social Support Questionnaire**

Norbeck JS , Lindsey AM , Carrieri VL . The development of an instrument to measure social support. Nurs Res 1981;30(5):264–9. 10.1097/00006199-198109000-00003 7027185

La Roche MJ , Turner C , Kalick SM . Latina mothers and their toddlers’ behavioral difficulties. Hisp J Behav Sci 1995;17(3):375–84. 10.1177/07399863950173007

**Post Traumatic Growth Inventory**

Tedeschi RG , Calhoun LG . The Posttraumatic Growth Inventory: measuring the positive legacy of trauma. J Trauma Stress 1996;9(3):455–71. 10.1002/jts.2490090305 8827649

Mystakidou K , Tsilika E , Parpa E , Kyriakopoulos D , Malamos N , Damigos D . Personal growth and psychological distress in advanced breast cancer. Breast 2008;17(4):382–6. 10.1016/j.breast.2008.01.006 18455402

Leiva-Bianchi M , Araneda A . Confirmatory factor analysis of the Post-Traumatic Growth Inventory after the Chilean earthquake. J Loss Trauma 2015;20(4):297–305. 10.1080/15325024.2013.873223

Mack J , Herrberg M , Hetzel A , Wallesch CW , Bengel J , Schulz M ,  The factorial and discriminant validity of the German version of the Post-traumatic Growth Inventory in stroke patients. Neuropsychol Rehabil 2015;25(2):216–32. 10.1080/09602011.2014.918885 24885533

Saltzman BM , Frank JM , Slikker W , Fernandez JJ , Cohen MS , Wysocki RW . Clinical outcomes of proximal row carpectomy versus four-corner arthrodesis for post-traumatic wrist arthropathy: a systematic review. J Hand Surg Eur Vol 2015;40(5):450–7. 10.1177/1753193414554359 25294736

**Psychological Capital Questionnaire**

Avey JB , Luthans F , Jensen SM . Psychological capital: a positive resource for combating employee stress and turnover. Hum Resour Manage 2009;48(5):677–93. 10.1002/hrm.20294

Luthans F , Youssef CM . Positive workplaces. Oxford handbook of positive psychology, 2nd edition. Oxford library of psychology. New York (NY): Oxford University Press; 2009. p. 579–88.

Wang Y , Chang Y , Fu J , Wang L . Work-family conflict and burnout among Chinese female nurses: the mediating effect of psychological capital. BMC Public Health 2012;12(1):915. 10.1186/1471-2458-12-915 23107113PMC3585697

**Psychological Well-being Questionnaire**

Ryff CD , Keyes CLM . The structure of psychological well-being revisited. J Pers Soc Psychol 1995;69(4):719–27. 10.1037/0022-3514.69.4.719 7473027

**Resilience Scale for Adults**

Friborg O , Hjemdal O , Rosenvinge JH , Martinussen M . A new rating scale for adult resilience: what are the central protective resources behind healthy adjustment? Int J Methods Psychiatr Res 2003;12(2):65–76. 10.1002/mpr.143 12830300PMC6878238

Friborg O , Barlaug D , Martinussen M , Rosenvinge JH , Hjemdal O . Resilience in relation to personality and intelligence. Int J Methods Psychiatr Res 2005;14(1):29–42. 10.1002/mpr.15 16097398PMC6878482

Friborg O , Martinussen M , Rosenvinge JH . Likert-based vs. semantic differential-based scorings of positive psychological constructs: a psychometric comparison of two versions of a scale measuring resilience. Pers Individ Dif 2006;40(5):873–84. 10.1016/j.paid.2005.08.015

Jowkar B , Friborg O , Hjemdal O . Cross-cultural validation of the Resilience Scale for Adults (RSA) in Iran. Scand J Psychol 2010;51(5):418–25. 10.1111/j.1467-9450.2009.00794.x 20149146

Hjemdal O , Friborg O , Braun S , Kempenaers C , Linkowski P , Fossion P . The Resilience Scale for Adults: construct validity and measurement in a Belgian sample. Int J Test 2011;11(1):53–70. 10.1080/15305058.2010.508570

Hilbig J , Viliūnienė R , Friborg O , Pakalniškienė V , Danilevičiūtė V . Resilience in a reborn nation: validation of the Lithuanian Resilience Scale for Adults (RSA). Compr Psychiatry 2015;60:126–33. 10.1016/j.comppsych.2015.02.003 25796289

Hjemdal O , Roazzi A , Dias MG , Friborg O . The cross-cultural validity of the Resilience Scale for Adults: a comparison between Norway and Brazil. BMC Psychol 2015;3(1):18. 10.1186/s40359-015-0076-1 26090106PMC4471935

**Resistance to Trauma Test**

Urra Portillo J , Escorial Martín S , Martínez Arias R . Development and psychometric properties of the Resistance to Trauma Test (TRauma). Psicothema 2014;26(2):215–21. 10.7334/psicothema2013.128 24755023

**Response to Stressful Experiences Scale**

Johnson DC , Polusny MA , Erbes CR , King D , King L , Litz BT ,  Development and initial validation of the Response to Stressful Experiences Scale. Mil Med 2011;176(2):161–9. 10.7205/MILMED-D-10-00258 21366078

**Sense of Coherence Scale**

Eriksson M , Lindström B . Validity of Antonovsky’s sense of coherence scale: a systematic review. J Epidemiol Community Health 2005;59(6):460–6. 10.1136/jech.2003.018085 15911640PMC1757043

**Solution-Focused Inventory**

Grant AM , Cavanagh MJ , Kleitman S , Spence G , Lakota M , Yu N . Development and validation of the Solution-Focused Inventory. J Posit Psychol 2012;7(4):334–48. 10.1080/17439760.2012.697184

Yang H , Hai T . Reliability and validity of the Chinese version of the solution‐focused inventory in college students. J Multicult Couns Devel 2015;43(4):305–15. 10.1002/jmcd.12023

**Values in Action**

Macdonald C , Bore M , Munro D . Values in action scale and the big 5: an empirical indication of structure. J Res Pers 2008;42(4):787–99. 10.1016/j.jrp.2007.10.003

Azañedo CM , Fernández-Abascal EG , Barraca J . Character strengths in Spain: validation of the Values in Action Inventory of Strengths (VIA-IS) in a Spanish sample. Clin Salud 2014;25(2):123–30. 10.1016/j.clysa.2014.06.002

Choubisa R , Singh K . Psychometrics encompassing VIA-IS: a comparative cross cultural analytical and referential reading. J Indian Acad Appl Psychol 2011;37(2):325–32.

Duan W , Bai Y , Tang X , Siu PY , Ho S . Virtues and positive mental health. Ment Health (Lond) 2012;38(2):1–8.

Duan W , Ho SM , Yu B , Tang X , Zhang Y , Li T ,  Factor structure of the Chinese Virtues Questionnaire. Res Soc Work Pract 2012;22(6):680–8. 10.1177/1049731512450074

Khumalo IP , Wissing MP , Temane QM . Exploring the validity of the Values-In-Action Inventory of Strengths (VIA-IS) in an African context. J Psychol Afr 2008;18(1):133–42. 10.1080/14330237.2008.10820180

Littman-Ovadia H . The effect of client attachment style and counselor functioning on career exploration. J Vocat Behav 2008;73(3):434–9. 10.1016/j.jvb.2008.08.004

Otake K , Shimai S , Ikemi A , Utsuki N , Peterson C , Seligman ME . [Development of the Japanese version of the Values In Action Inventory of Strengths (VIA-IS)]. Shinrigaku Kenkyu 2005;76(5):461–7. 10.4992/jjpsy.76.461 16447695
